# Improving the Diagnosis of Vulvovaginitis: Perspectives to Align Practice, Guidelines, and Awareness

**DOI:** 10.1089/pop.2020.0265

**Published:** 2020-10-16

**Authors:** Haywood Brown, Madeline Drexler

**Affiliations:** ^1^ Professor Obstetrics Gynecology, University South Florida, Tampa, Florida, USA.; ^2^ Harvard Public Health, Harvard T.H. Chan School of Public Health, Boston, Massachusetts, USA.

**Keywords:** vulvovaginitis, adverse reproductive consequences, bacterial vaginosis, *Candida* infections, trichomoniasis, interventions, guidelines

## Abstract

Vulvovaginitis is a frequent reason for women to see a health care provider and has been linked to adverse reproductive and psychosocial consequences. Accurate diagnosis is a cornerstone of effective treatment, yet misdiagnosis of this condition approaches 50%, raising the risk of recurrence. The past 3 decades have seen few improvements over the traditional means of diagnosing the 3 main causes of vaginitis: bacterial vaginosis, *Candida* infections, and trichomoniasis. Newer molecular tests, which are both more sensitive and specific, have introduced the potential to transform the diagnosis of vaginitis—ensuring more accurate diagnoses and timely interventions, while reducing health care costs and enhancing patients' quality of life. Clinical approaches and professional guidelines should be updated to reflect advances in molecular testing and improve the diagnosis and management of acute and recurrent vulvovaginitis.

## Introduction

Vulvovaginitis, or vaginitis, is frequently cited as the most common reason that women visit their primary care providers for a gynecologic-related diagnosis in the United States.^1^ Vaginal symptoms negatively impact patients in terms of discomfort and pain, days lost from school, work, sexual functioning, and self-image.^2,3^

Defined as inflammation or infection of the vagina, vaginitis presents a spectrum of symptoms, including itching, burning, irritation, dyspareunia, vaginal odor, and abnormal vaginal discharge.^3^ The most common causes are bacterial vaginosis (BV), vulvovaginal candidiasis (VVC), and trichomoniasis (TV).^4^ Among patients with vaginal symptoms, BV is diagnosed in 22%–50% of cases, VVC in 17%–39% of cases, and TV in 4%–35% of cases. Vaginitis also may remain undiagnosed in 7%–72% of patients.^3,5^ Vaginitis is responsible for more than 10 million office visits annually.^6^ In the United State, the estimated cost of treating symptomatic BV—the most common form of vaginal infections—approaches $1.3 billion.^7^ That figure nearly triples when the costs of reproductive health consequences—such as BV-associated surgical site infections—are considered, and also is affected by the financial implications of preterm births and health care costs for women with the human immunodeficiency virus (HIV).^7^

### Bacterial vaginosis

BV represents a change in the normal vaginal microbiome and is not a true infectious or inflammatory state.^3,8,9^ BV is characterized by a change in the vaginal microbiota dominated by the *Lactobacillus* species to a polymicrobial anaerobe-dominated microbiota that includes *Gardnerella vaginalis*, *Atopobium vaginae*, *Prevotella*, *Bacteroides*, *Peptostreptococcus*, *Mobiluncus*, *Sneathia* (*Leptotrichia*), *Mycoplasma*, and BV-associated bacteria.^10^ Though many patients with BV are asymptomatic, those who present with symptoms commonly report abnormal vaginal discharge and odor, often following vaginal intercourse and menses.^5^

BV can have serious health repercussions and raises the risk of contracting a number of sexually transmitted infections (STIs).^11,12^ For this reason, all women with suspected BV should be evaluated for HIV and other STIs.^13^ BV is the most common cause of abnormal vaginal discharge in patients of reproductive age and has a higher prevalence in Black, Hispanic, and Mexican American women, compared with non-Hispanic white women.^3,14^ The higher prevalence among non-Hispanic Black women has been theorized to contribute to observed racial disparities in rates of preterm births.^5^

### Vulvovaginal candidiasis

VVC represents inflammation and infection of the vagina with the *Candida* species. It is the second most common cause of vaginitis, after BV.^9^ VVC is most commonly caused by *C. albicans*, but also may be triggered by other *Candida* species or yeasts, including *Candida glabrata*.^15^ An estimated 75% of women will have at least 1 episode of VVC during their lifetime, and 40%–45% will experience 2 or more episodes.^9^

### Trichomoniasis

Vaginal TV, which is caused by infection with the protozoan parasite *Trichomonas vaginalis*, is the most common nonviral STI in the United States, with approximately 3–5 million cases annually.^3,16^ In 2015, there were 139,000 initial physician office visits in the United States for TV, a number that has been fairly stable since the 1990s.^13^

Health disparities persist in the epidemiology of *T. vaginalis* infections. African American women are 10 times more commonly affected, compared with non-Hispanic white women.^3,17^ National Health and Nutrition Examination Survey data from 2013–2016 indicated an overall *Trichomonas vaginalis* infection prevalence of 2.1% among women ages 14–59 years, with the highest prevalence rate of 9.6% among African American women, 1.4% for Hispanic women, and 0.8% for non-Hispanic white women.^18^

More than 50% of patients with TV are asymptomatic or have minimal symptoms; symptomatic patients with TV may report abnormal vaginal discharge, itching, burning, or postcoital bleeding.^17^

*T. vaginalis* infection is associated with 2–3 times increased risk for HIV acquisition, preterm birth, and other adverse pregnancy outcomes among pregnant women. Among women with HIV infection, *T. vaginalis* infection is associated with increased risk for pelvic inflammatory disease. Routine screening for *T. vaginalis* is recommended in asymptomatic women with HIV infection.^9^

### Coinfections

Coinfections are common in vaginitis, making accurate diagnosis and treatment of the 3 most common microbial pathogens challenging. More than 20% of infectious vaginitis cases may be mixed.^19,20^

In a recent study of an investigational molecular diagnostic assay, Schwebke et al found that coinfection rates by 2 or more organisms were 20% by reference testing and approximately 25% by investigational testing. Using reference methods, BV alone was detected in 31.9% of the total subjects, the *Candida* species group alone in 13.9%, and TV alone in 1.8%; in 31.1% of subjects, no infection was found.^19^ A different analysis of a molecular diagnostic assay found that women with BV alone or with concurrent *Candida* spp. infections had high rates of coinfection—24.4%–25.7%—with STIs.^20^

## Traditional Diagnostic Methods

In the clinician's office, the causes of vaginal symptoms might be determined by pH, a potassium hydroxide (KOH) test, and microscopic examination of fresh samples of the discharge. An elevated pH (ie, ≥4.5) is common with BV or TV. The absence of trichomonads in saline, or fungal elements in KOH samples, does not rule out these infections because the sensitivity of microscopy is approximately 50% compared with more recent nucleic acid amplification tests (NAATs).^3,9^

### Bacterial vaginosis

Traditionally, the diagnosis of BV is based on microscopy and clinical symptoms, as reflected in Amsel criteria^21^ and the Nugent scoring system, which assigns a value to different bacterial morphotypes seen on Gram stain of vaginal secretions.^22^ The diagnosis is based on the presence of 3 of the following 4 Amsel criteria^9,21^:

1. Discharge: homogeneous, thin, white-gray discharge that smoothly coats the vaginal walls
2. Clue cells: more than 20% clue cells on saline microscopy
3. pH >4.5: vaginal fluid pH >4.5
4. Positive KOH: positive KOH whiff test result

Detection of 3 of 4 Amsel criteria has been correlated with results by Gram stain with scoring, which is considered the reference standard. Gram stain—which determines the relative concentrations of lactobacilli, Gram-negative and Gram-variable rods and cocci, and curved Gram-negative rods—is considered the reference-standard laboratory method for diagnosing BV.^9^ In clinical settings, however, Gram stain with Nugent scoring is often impractical, and Amsel criteria typically are used for the diagnosis of BV.^3^ Amsel clinical criteria have a reported sensitivity of 92% and a specificity of 77% compared with Gram stain with Nugent scoring.^23^

Although Amsel criteria are more specific, the Nugent score is more sensitive; the tests concur in 80%–90% of cases. One advantage of Nugent scoring is high intraobserver and interobserver reliability and reproducibility. Clue cells correlate best with Gram stain results and are thought to be the most reliable feature in making a diagnosis of BV. A vaginal pH >4.5 is considered the most sensitive criterion.^8^

### Vulvovaginal candidiasis

Culture for yeast is the reference standard for diagnosing VVC.^9^ Examination of a wet mount with KOH preparation should be performed for all women with symptoms or signs of VVC, and women with a positive result should be treated. Microscopy also may be limited by self-treatment before evaluation, making it more difficult for the health care provider to visualize yeast on microscopy.^24^ Culture- and polymerase chain reaction-based tests offer alternatives for negative wet mounts or complex cases.^9^ Importantly, failed medical therapy for clinically suspected yeast infections and recurrent yeast may be related to resistance associated with the *Candida* species.^25^

### Trichomoniasis

Before molecular detection methods became available, culture was considered the standard method for diagnosing *T. vaginalis* infection. Culture has a sensitivity of 75%–96% and a specificity of up to 100%.^26^ In practice, the most common method for *T. vaginalis* diagnosis might be microscopic evaluation of wet preparations of genital secretions because of convenience and relatively low cost. In vaginal specimens, however, the sensitivity of wet mount is low (51%–65%).^9,26^ The absence of trichomonads in saline or fungal elements in KOH samples does not rule out a TV infection.^3,9^

### Dangers of misdiagnosis

Because vaginitis is a global term for a nonspecific syndrome, and because the condition has 3 distinct etiologies (ie, BV, VVC, TV) with 3 different groups of causative organisms, accurate differential diagnosis is essential for effective treatment. Clinicians cannot rely on symptoms alone to distinguish confidently between the causes of vaginitis.^6^

As molecular tests continue to become commercially available, the limitations of microscopy in diagnosing the specific causes of vaginitis have become more salient. In one study, the sensitivity of microscopy was 22% for the *Candida* species and 62% for *Trichomonas vaginalis*, compared with Gram stain.^27^ In a more recent report, the sensitivity of microscopy was 50% compared with a standard of NAAT for TV or culture for the *Candida* species.^27,28^

Traditional laboratory methods such as Gram stain and culture may be highly subject to sampling, transport conditions, and technical proficiency, and may have prolonged turnaround times.^20^ Traditional in-clinic diagnostic methods often can lead to inaccurate or incomplete diagnoses and, in some cases, high recurrence rates. Because current standards of care rely on the microscopic evaluation of vaginal samples and an empiric diagnosis, the approach relies on the clinician's level of training and often can result in incorrect diagnosis and treatment.^29,30,31^ Clinical diagnosis using Amsel criteria and laboratory diagnosis using Nugent criteria also involve subjective components, and approximately half of symptomatic women evaluated for vaginal infections are not diagnosed accurately when using conventional testing approaches (Amsel plus wet mount).^32^

In a 2020 study, Schwebke et al summarize problems associated with traditional diagnostic methods for vaginitis. Among these: lack of equipment in the clinic, subjectivity of the clinical end points used and inconsistent application of these end points between practitioners, lack of proper training in microscopy, and overall poor sensitivity of the tests themselves.^19,33^ Diagnosis of the underlying infectious causes of vaginitis is further complicated by the common symptomatology reported for BV, VVC, and TV,^9,34^ the incidence of mixed infections or coinfections,^35^ and the recurrence of vaginal symptoms.^36,37^ Considered together, the authors conclude, “these barriers result in many women being misdiagnosed based on nonspecific observations, leading to incorrect, misguided, or prolonged treatment.”^19^

## The Arrival of Molecular Diagnostics

Recently, commercially available molecular diagnostic tests have been shown to have superior sensitivity and specificity, compared with conventional clinical assessment, in diagnosing the common infectious causes of vaginitis.

NAAT provides the ability to aid in the detection of several bacterial species implicated in BV, including *Gardnerella* and *Lactobacillus* (the latter of which may help prevent infection by producing lactic acid, hydrogen peroxide, bacteriocins, or through competitive exclusion of other bacteria).^38^ Relative levels of these species are evaluated to determine a qualitative (positive or negative) result. Earlier nucleic-acid-based technologies do not rely on amplified probes but rather on direct probe binding and detection of the target (ie, BD Affirm, Becton Dickinson, Sparks, Maryland)—an important distinction between direct detection and amplified probe technologies.^39^ The superiority of NAAT methodologies compared with direct probes has been demonstrated extensively.^29,32^ Molecular techniques offer a distinct advantage over traditional methods in diagnosing bacterial vaginosis.

Microbes that commonly cause BV, such as *Gardnerella*, *Atopobium*, and *Prevotella*, are present in women both with and without BV as currently defined—meaning that detection alone does not provide adequate specificity. Multiplexed amplified molecular methods—in which multiple microbes can be accurately quantitated at very high numbers (106 CFU/ml or more)—allow the retraction of lactobacilli and overgrowth of *Gardnerella*, *Atopobium*, and other microbes in BV to be objectively measured, analyzed, and assessed.^19^

Molecular techniques offer a distinct advantage over traditional methods in diagnosing BV.^19^ Given the low predictive value of current clinical practice, many women are misdiagnosed and require multiple medical appointments before resolving their BV symptoms. A NAAT offers better analytical and clinical performance than the current standard of care. Data from a multicenter study show that a 3-target NAAT had high sensitivity, specificity, and negative and positive predictive values (98.7%, 95.9%, 92.9%, and 96.9%, respectively).^31^

A prospective multicenter clinical study was conducted to validate the performance of 2 in vitro diagnostic transcription-mediated amplification NAATs for the diagnosis of BV, VVC, and TV. Patient- and clinician-collected vaginal-swab samples obtained from women with symptoms of vaginitis were tested with the Aptima BV and Aptima Candida/Trichomonas vaginitis assays. The results were compared with Nugent (plus Amsel for intermediate Nugent) scores for BV, *Candida* cultures and DNA sequencing for VVC, and a composite of NAAT and culture for *Trichomonas vaginalis*.^19^

In this study, the prevalence of infection was similar for clinician- and patient-collected samples: 49% for BV, 29% for VVC caused by the *Candida* species group, 4% for candidiasis caused by *Candida glabrata*, and 10% for *T. vaginalis*. Sensitivity and specificity estimates for the tests in clinician-collected samples were 95.0% and 89.6%, respectively, for BV; 91.7% and 94.9% for the *Candida* species group; 84.7% and 99.1% for *C. glabrata*; and 96.5% and 95.1% for *T. vaginalis*. Sensitivities and specificities were similar in patient-collected samples. In a secondary analysis, clinicians' diagnoses, in-clinic or point-of-care assessments, and results were compared with standard reference methods. Overall, the molecular assays provided improved sensitivity and specificity compared with clinicians' diagnoses and in-clinic assessments, demonstrating that the molecular assays more accurately predict infection than do traditional diagnostic methods.^19^

## Challenges of Nonmolecular Platforms

In-office point-of-care tools, such as microscopy, wet mount, and KOH represent common elements of the traditional evaluation, but often lack diagnostic accuracy for candida, TV, and vulvovaginitis that molecular tools provide.

Yeast culture is used routinely to provide species-level identification in diagnosing candidiasis, particularly for less common species of yeast that can cause infection. But there are additional diagnostic considerations. Accurate identification of azole-resistant species, such as *C. glabrata*, is also critical in guiding appropriate treatment.^19^ And although microscopy has been regarded as cost-effective for use in clinical practice, its sensitivity for *C. albicans* is approximately 50%–70%, resulting in a substantial percentage of patients with symptomatic VVC being misdiagnosed.^40^

Current practice guidelines from the American College of Obstetricians and Gynecologists (ACOG), the Centers for Disease Control and Prevention (CDC), and the Infectious Disease Society of America all recommend NAAT as the reference standard for diagnosis of TV. Yet in practice, diagnosing TV remains a challenge.^41^ Microscopy, historically the most common diagnostic modality, has poor sensitivity for TV. In a recent retrospective cohort study of women who delivered over a 2-year period at 1 institution, testing for *Trichomonas vaginalis* infection was conducted by wet mount microscopy or by NAAT for routine prenatal testing or symptomatic visits. The sensitivity for microscopy compared with NAAT was 26%, with a specificity of 99%.^41^

Highly sensitive and specific tests are recommended for detecting *T. vaginalis.*^9^ Among women, NAAT is highly sensitive, often detecting 3–5 times more *T. vaginalis* infections than wet mount microscopy, a method with poor sensitivity (51%–65%).^42,43^ There are several Food and Drug Administration (FDA)-cleared NAATs for the detection of trichomonas in women, including the Aptima Trichomonas vaginalis assay (Hologic Gen-Probe, San Diego, CA) and the BD ProbeTec and BDMax assay (Becton Dickinson, Sparks, Maryland).^9,44^

## The Presence of Coinfections

The presence of coinfections makes accurate diagnosis of vaginitis even more challenging—and more urgent. In a multicenter clinical trial, researchers observed less accurate clinician diagnosis of BV, based on clinical observations, when *Trichomonas vaginalis* and/or *Candida* spp. also were detected by the trial reference methods, compared with when BV alone was detected. Sensitivity of the Amsel criteria in women with BV decreases when *Trichomonas vaginalis* and/or *Candida* spp. are present.^45^

A diagnostic test using NAAT, if available, is strongly advised if BV or TV is suspected.^30^ Gaydos et al note that if microscopy is negative but yeast is suspected, additional testing by culture or NAAT for the *Candida* species is important, because microscopy is not sufficiently sensitive to exclude *Candida* in symptomatic patients. Schwebke et al note that multiplex capabilities, using a single vaginal swab, allow sensitive and specific differential diagnoses for BV, VVC, and TV, regardless of coinfection status.^19^

Use of NAAT facilitates the detection of numerous vaginitis-causing pathogens—including those for BV, *Candida* spp., *Chlamydia trachomatis* (CT), *Neisseria gonorrhoeae*, and trichomonas—from a single collected specimen. In a retrospective assessment, Van Der Pol et al showed that a large percentage (>85%) of individuals positive for any STI also were positive for BV or *Candida* spp. Women who were positive for BV were significantly more likely to have a CT infection, a trichomonas infection, or any STI. Indeed, it was common for women to have multiple pathogens that may play a role in vaginitis (including STI). The authors concluded that a diagnostic tool that determines only a single pathogen or syndrome likely underdiagnoses STI infections that each require specific clinical management.^20^

## The Context of Testing

Practice patterns exert a strong influence on vulvovaginitis diagnosis. Nyirjesy has noted that because women with chronic or recurrent symptoms present a therapeutic challenge for health care providers, vulvovaginitis may be ignored or trivialized and remain unresolved for months or years. As a result, many women self-treat, resorting to over-the-counter and alternative medicines—in some cases, exacerbating symptoms and making the problem worse.^46^

Women also seek care from a variety of provider types or clinical settings. For symptomatic women, diagnostic testing often depends on the clinic type, available services, and provider assumptions. These real-world factors may restrict diagnostic testing of certain causes of vaginitis. Making this type of testing both simple and available could both improve services for women and reduce the stigma associated with STI testing.^20^

With superior sensitivity for 3 of the most common causes of vulvovaginitis, NAAT represents an efficient test methodology that can be derived from a single sample and processed individually or in parallel. Paladine has noted that newer laboratory tests such as DNA and antigen testing for BV and VVC, or vaginal fluid sialidase testing for BV, may have similar or better sensitivity and specificity compared with traditional office-based testing.^34^ In theory, improvements in clinicians' microscopy skills should result in better outcomes and minimize the diagnostic limitations of microscopy; as discussed elsewhere in this paper, microscopy training and improved use of empiric skills may not be enough to reliably discern coinfection and other conditions.

Although the literature regarding cost is incomplete, it has been argued that the use of NAAT may incur expenses greater than those for clinical evaluation and microscopy, and also may produce false-positive results in patients with low pretest probabilities of infection.^47^

Given these considerations, some clinicians have suggested that, in routine cases of vulvovaginitis, it may be reasonable to treat based on microscopy results if microscopic diagnostic criteria are confirmed, reserving NAAT for patients at risk of TV or patients who have resistant or recurrent symptoms.^47^ Others have concluded that reserving molecular tests for complicated cases and emphasizing improved microscopy and clinical evaluation may represent the most cost-effective path forward, or have pointed to efficiencies from empiric treatment resulting from clinical evaluation supplemented with molecular diagnostics.^32,34^

Comparative cost-effectiveness data are just beginning to emerge; analyses that compare the costs of newer technologies with microscopy, and the costs of new technologies with each other, are limited; additional information will be required to more fully inform clinical utility.^32,34^

## Quality of Life

Vulvovaginitis may significantly impact a woman's quality of life, self-esteem, self-image, productivity, and sexual health.^2,48^

A cross-sectional online survey conducted among women who reported 4 or more yeast infections over 12 months in 5 European countries and the United States looked at health-related quality of life (HRQoL). The study found that subjective health status and HRQoL during and in between acute episodes in women with recurrent yeast infections were significantly worse than in the general population. The average index score in women with chronic infections was comparable to that of other chronic afflictions such as asthma or chronic obstructive pulmonary disease.^49^

In a 2017 report, Chavoustie et al found that BV represents a highly stressful condition that may take a serious emotional toll, negatively impacting self-image, social and physical activities, and diminishing productivity at work and/or school.^50^

## Current Practice: Varied Diagnostic Approaches

As the science continues to advance, providers and professional societies should evaluate existing best practices for diagnosing vaginitis. To understand current diagnostic practices, Nyirjesy et al conducted a survey among 333 physicians to measure awareness of vaginitis clinical guidelines and use of in-office, point-of-care, and molecular diagnostic tools. Coupled with a chart review of more than 900 patients, the study found significant discrepancies between guideline recommendations and clinical practices.^51^

Physicians were most familiar with guidelines for VVC and BV; fewer than half were familiar with those for TV. The study also revealed that while access to point-of-care tools used to evaluate and diagnose vaginitis varies by practice, there was limited access to all 3 tools (microscope, pH test strips, KOH solution) required to perform a full Amsel workup for a BV diagnosis (47% obstetricians/gynecologists vs. 32% primary care physicians).

**FIG. 1.**
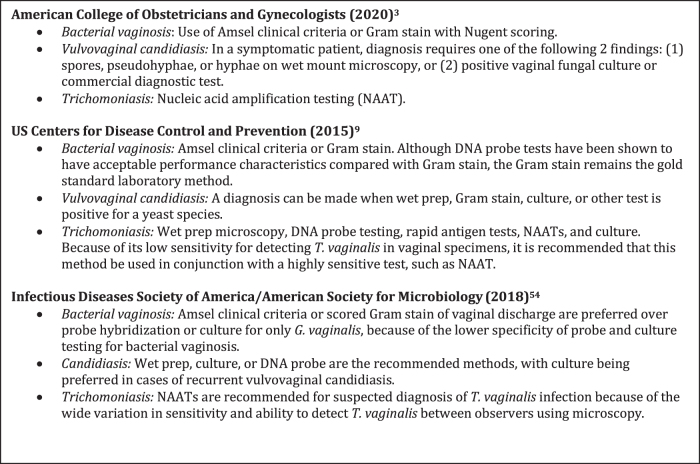
Practice Guidelines for Diagnosing Vaginitis.

Based on guidelines, only 66% of patients evaluated for VVC, 45% of patients evaluated for BV, and 17% evaluated for TV received an optimal workup. Among TV-positive patients, 75% received chlamydia/gonorrhea testing, 42% were tested for HIV/AIDS, partner therapy was noted in 59% of cases, and 47% returned to be retested within 3 months (per guidelines) (Fig. 1).

A clinical study by Hillier et al of 303 women presenting with vaginitis symptoms adds further evidence that clinical workups often deviate from professional guidelines, resulting in many symptomatic women receiving inappropriate treatment. In this community practice setting, standard point-of-care tests—including vaginal pH, KOH/whiff, and wet mount microscopy—were rarely performed. Indeed, 42% of symptomatic women received inappropriate treatment; women who received empiric treatment were more likely to have recurrent visits within 90 days.^52^

These studies offer evidence that professional guidelines need to integrate clinician behavior with advances in technology, and evolve to include lab-based assessments to ensure optimal and accurate treatment for patients with vulvovaginitis.^49^ Current ACOG guidelines, which acknowledge that appropriate office-based tools may not always be available, allow for the use of commercial tests that have been approved by the FDA for the diagnosis of vulvovaginitis in such circumstances.^3^ Payer policies for vulvovaginitis must similarly evolve to cover the use of these tests, reducing any cost burden to patients.

## The Path Forward

Until the recent introduction of molecular tools, there have been few diagnostic advances over the last 3 decades—a signal that vulvovaginitis continues to be a relatively neglected area of medical research.^50^

In an article published a full decade ago—with the stark and succinct title “Diagnosing Vaginal Infections: It's Time to Join the 21st Century”—Van Der Pol argued that health care's continued reliance on diagnostic methods with poor performance stems in part from the lack of a public health mandate to reduce the burden imposed by vaginal infections. The result is a continued lack of guidelines, access, and coverage for more sensitive and specific diagnostic assays. According to Van Der Pol, “A call to action from all professionals involved in improving women's reproductive and sexual health is required if we are to successfully join the 21st century and begin to improve diagnosis and reduce negative outcomes associated with vaginal diseases.”^53^ As the evidence in this section attests, it is time for current clinical practice and professional guidelines to evolve with current science in diagnostics and address gaps in clinical practice.

## Next Steps

Given today's rapidly evolving diagnostic landscape, how can medicine better align clinician assessment with updated and simplified algorithms for patient care? How should highly sensitive molecular diagnostics be leveraged to improve the health and well-being of women with acute or recurrent vaginitis?

The availability of sensitive and specific molecular tools requires a review of the existing diagnostic approaches. To be sure, molecular diagnostics will be more applicable to some of the causes of vaginitis than to others. When a microscope is available, visible VVC and TV organisms permit diagnosis in a physician's office. By contrast, diagnosis of BV—a condition that involves multiple organisms, and one that often relies on subjective clinical judgment—will become more accurate using NAAT.

Molecular testing also may facilitate new possibilities for diagnostic assessment as the data for patient-collected samples become more robust. In the post-COVID-19 medical landscape, telehealth and “touchless testing” are expected to remain prominent; indeed, for many women with suspected vaginitis, self-collection of vaginal specimens, whether at home or in a physician's office, may further transform the diagnosis and treatment of vaginitis.

Professional guidelines must be aligned to accommodate the use of amplified molecular diagnostics and to recognize that a new reference standard has emerged. These professional guidelines—from the ACOG, the American Academy of Family Physicians, and the CDC—must evolve to reduce confusion and to improve differential diagnosis.

What is at stake for women in this new diagnostic environment? By moving toward highly sensitive molecular diagnostics, and more streamlined care with faster laboratory turnaround, patients can be provided with not only a greater sense of satisfaction with their medical care but also a greater sense of empowerment. The ability of patients to self-collect specimens for analysis with NAAT may provide an opportunity to lower costs by reducing office visits and shifting to telemedicine visits for follow-up. Reliable and accurate diagnosis of vulvovaginitis, a condition long neglected in clinical medicine, is possible.

The next phase of women's health medicine will be built on a foundation of advanced molecular diagnostics and will rely on appropriate use of the most up-to-date tools. The challenge is to ensure that clinical skill sets and professional guidelines keep pace with these important advances.

## Appendix A1. Suggested Reading

- Allen-Davis JT, Beck A, Parker R, Ellis JL, Polley D. Assessment of vulvovaginal complaints: accuracy of telephone triage and in-office diagnosis. Obstet Gynecol 2002;99:18–22.
- Anderson MR, Klink K, Cohrssen A. Evaluation of vaginal complaints. JAMA 2004;291:1368–1379.
- Bilardi J, Walker S, McNair R, et al. Women's management of recurrent bacterial vaginosis and experiences of clinical care: a qualitative study. PLoS One 2016;11:e0151794.
- Campbell L, Woods V, Lloyd T, et al. Evaluation of the OSOM Trichomonas rapid test versus wet preparation examination for detection of *Trichomoas vaginalis* vaginitis in specimens from women with a low prevalence of infection. J Clin Microbiol 2008;46:3467–3469.]
- Carr PL, Rothberg MB, Friedman RH, Felsenstein D, Pliskin JS. “Shotgun” versus sequential testing. Cost-effectiveness of diagnostic strategies for vaginitis. J Gen Intern Med 2005;20:793–799.
- Cartwright CP, Lembke BD, Ramachandran K, et al. Development and validation of a semiquantitative, multitarget PCR assay for diagnosis of bacterial vaginosis. J Clin Microbiol 2012;50:2321–2329.
- Cartwright C, Lembke B, Ramachandran K, et al. Comparison of nucleic-acid amplification assays with BD affirm VPIII for the diagnosis of vaginitis/vaginosis in symptomatic women. J Clin Microbiol 2013;51:3694–3699.
- Cherpes TL, Wiesenfeld HC, Melan MA, et al. The associations between pelvic inflammatory disease, *Trichomonas vaginalis* infection, and positive herpes simplex virus type 2 serology. Sex Transm Dis 2006;33:747–752.
- Gatski M, Martin DH, Clark RA, Harville E, Schmidt N, Kissinger P. Co-occurrence of *Trichomonas vaginalis* and bacterial vaginosis among HIV-positive women. Sex Transm Dis 2011;38:163–166.
- Hartmann AA. [*Gardnerella vaginalis* infection. Clinical aspects, diagnosis and therapy]. Urologe A 1987;26:252–255.
- Huppert JS, Batteiger BE, Braslins P, et al. Use of an immunochromatographic assay for rapid detection of *Trichomonas vaginalis* in vaginal specimens. J Clin Microbiol 2005;43:684–687.
- Huppert JS, Mortensen JE, Reed JL, et al. Rapid antigen testing compares favorably with transcription-mediated amplification assay for the detection of *Trichomonas vaginalis* in young women. Clin Infect Dis 2007;45:194–198.
- Lejeune M. Improving cost and clinical efficacy of vaginitis detection and management via an FDA-authorized nucleic acid amplification test: an economic model. Am J Obstet Gynecol 2017;217:738 (IDSOG Abstract).
- Lowe NK, Neal JL, Ryan-Wenger NA. Accuracy of the clinical diagnosis of vaginitis compared with DNA probe laboratory standard. Obstet Gynecol 2009;113:89–95.
- Nelson DB, Hanlon A, Hassan S, et al. Preterm labor and bacterial vaginosis-associated bacteria among urban women. J Perinat Med 2009;37:130–134.
- Nyirjesy P, Seeney SM, Grody MH, Jordan CA, Buckley HR. Chronic fungal vaginitis: the value of cultures. Am J Obstet Gynecol 1995;173:820–823.
- Oleen-Burkey MA, Hillier SL. Pregnancy complications associated with bacterial vaginosis and their estimated costs. Infect Dis Obstet Gynecol 1995;3:149–157.
- Pfaller MA. Application of culture-independent rapid diagnostic tests in the management of invasive candidiasis and cryptococcosis. J Fungi 2015;1:217–251.
- Rivers CA, Adaramola OO, Schwebke JR. Prevalence of bacterial vaginosis and vulvovaginal candidiasis mixed infection in a southeastern American STD clinic. Sex Transm Dis 2011;38:672–674.
- Sobel JD. Epidemiology and pathogenesis of recurrent vulvovaginal candidiasis. Am J Obstet Gynecol 1985;152:924–935.
- Tenove FC, Baron EJ, Gaydos CA. Self-collected specimens for infectious disease testing. Clin Microbiol Newsl 2017;39:51–56.
- Thompson A, Timm K, Borders N, Montoya L, Culbreath K. Diagnostic performance of two molecular assays for the detection of vaginitis in symptomatic women. Eur J Clin Microbiol Infect Dis 2020;39:39–44.
- United Healthcare Community Plan Effective Date May 1, 2020. Genitourinary Pathogen Nucleic Acid Detection Panel Testing.
- van der Veer C, van Houdt R, van Dam A, de Vries H, Bruisten S. Accuracy of a commercial multiplex PCR for the diagnosis of bacterial vaginosis. J Med Microbiol 2018;67:1265–1270.
- Wiesenfeld HC, Macio I. The infrequent use of office-based diagnostic tests for vaginitis. Am J Obstet Gynecol 1999:181:39–41.
- Wiesenfeld HC, Hillier SL, Krohn MA, Landers DV, Sweet RL. Bacterial vaginosis is a strong predictor of *Neisseria gonorrhoeae* and *Chlamydia trachomatis* infection. Clin Infect Dis 2003;36:663–668.
